# A new modified limited incision for superficial parotidectomy compared to modified Blair’s incision

**DOI:** 10.1097/MS9.0000000000001123

**Published:** 2023-08-03

**Authors:** Omar Salem Khattab Alomar

**Affiliations:** Department of General Surgery, College of Medicine, University of Baghdad, Baghdad, Iraq

**Keywords:** cosmesis, facial nerve, modified incision, parotidectomy

## Abstract

**Background:**

Many incisions have been used to perform parotidectomy, but they result in a visible scar on the neck and may cause patient dissatisfaction by producing disfigurement sometimes.

**Aim of Study:**

The aim of this study is to use a modified limited incision for superficial parotidectomy as an alternative to the classical incision, with no obvious scarring and without affecting the identification of the facial nerve, resulting in better cosmesis.

**Patients and Methods:**

A prospective comparative study on 100 patients has undergone superficial parotidectomy using a modified limited incision and another 100 patients who have undergone superficial parotidectomy using the modified Blair’s incision (control group) for variant benign pathologies. The surgeries were conducted in four hospitals (three private and one public) in Baghdad, Iraq, from January 2016 to September 2022. In both groups, the patients were followed up through outpatient visits to assess the cosmetic result of the incision and detect postoperative complications.

**Results:**

All tumors were removed with no need for extending the skin incision. The cosmetic result of the incision was very satisfactory and only a nominal scar could be seen 6 months after surgery. Five percent of patients only developed postoperative complications.

**Conclusion:**

A modified limited incision for superficial parotidectomy provides better patient satisfaction compared to a modified Blair’s incision. The modified limited incision can be performed safely with a better cosmetic appearance of the surgical scar compared to the standard incision.

## Introduction

Highlights

- Many incisions have been used to perform parotidectomy.
- Most incisions result in a visible scar and may cause patient dissatisfaction.
- We used a modified limited incision as an alternative to classical incisions.
- There was no obvious scarring.

The operative treatment for parotid tumors ,whether malignant or benign, is parotidectomy. The commonly used incision is that described by Blair in 1912 (the lazy S incision) and modified by Bailey in 1941^1^. This incision provides adequate exposure but results in a visible scar on the neck and may cause patient dissatisfaction by producing cervical or facial disfigurement sometimes. Many incisions have been used to avoid the disfigurement of Blair’s incision, like the retroauricular hairline incision^2^, retroauricular^3^, periauricular^4,5^, and modified face-lift^6–9^.

Limited incisions have been used by many surgeons for small and medium-sized parotid tumors located in the superficial lobe, and in selected cases of total parotidectomies that were performed for benign and selected malignant tumors^5,10,11^. They can be safely performed without increasing the risk of facial nerve (FN) injury and other complications. However, these techniques remain confined to the hands of experienced surgeons and have some limitations^5,10,11^.

Recently, the esthetic consideration of the parotidectomy scar has been reviewed. The face-lift incision improves the surgical wound’s appearance compared to the classic bayonet-shaped incision by eliminating its cervical portion, although it retains a visible occipital scar^12,13^.

The life quality of numerous patients was adversely affected after parotidectomy when there was a noticeable scar^14^. The long visible scar can lead to long-term psychosocial complications to the patients, and many patients refused doing parotidectomy because of the visible facial scar, until the tumor grows into large size or becomes malignant. For this reason, we decided to use a modified limited incision for superficial parotidectomy that is safe and neither increasing the operating time nor the morbidity associated with parotidectomy.

The aim of this study is to assess the use of a modified limited incision for superficial parotidectomy as an alternative to the classical incision, with no obvious scarring and without affecting the identification of the FN, resulting in a better cosmesis.

## Patients and methods

A prospective comparative study was carried out at Baghdad Teaching Hospital (Medical City), and private hospitals in Baghdad, from January 2016 to September 2022. Patients included in this study were those with parotid tumors who had undergone superficial parotidectomy for benign pathologies. Exclusion criteria were if the tumor greater than 4 cm in diameter, deep lobe tumor, tumor fixation to the skin, tumor recurrence, FN palsy or the patient refused collaboration. Two hundred patients with tumors in the superficial lobe of the parotid gland were recruited according to the selected criteria. The patients were divided into two groups. Group A included 100 patients who underwent superficial parotidectomy using our modified limited incision, and group B (control group) included 100 patients who underwent superficial parotidectomy via the modified Blair’s incision.

The study sample size was determined by the Raosoft sample size calculator, considering a 95% CI and a 7% margin of error.

Ethical considerations were obtained according to the Helsinki Declaration. Written consent from the patient was obtained after explaining the operation and the possible complications. The approval of the ethics committee was obtained from the Health Ethics Committee in the College of Medicine, University of Baghdad, with a registration number: 44. The methods of this article were prepared according to Strengthening the Reporting of cohort, cross-sectional and case–control studies in Surgery (STROCSS) Criteria^15^. The research was registered at the research registry: https://www.researchregistry.com, with a unique identifying number researchregistry 8594.

After taking a history, a physical examination, and routine laboratory investigations. Ultrasonography of the parotid region was done followed by computed tomography scan, MRI, and fine needle aspiration cytology. These investigations were done to all patients to identify the benign from malignant tumors, and to be sure about the location of the tumor to exclude deep lobe tumors.

**Figure 1:**
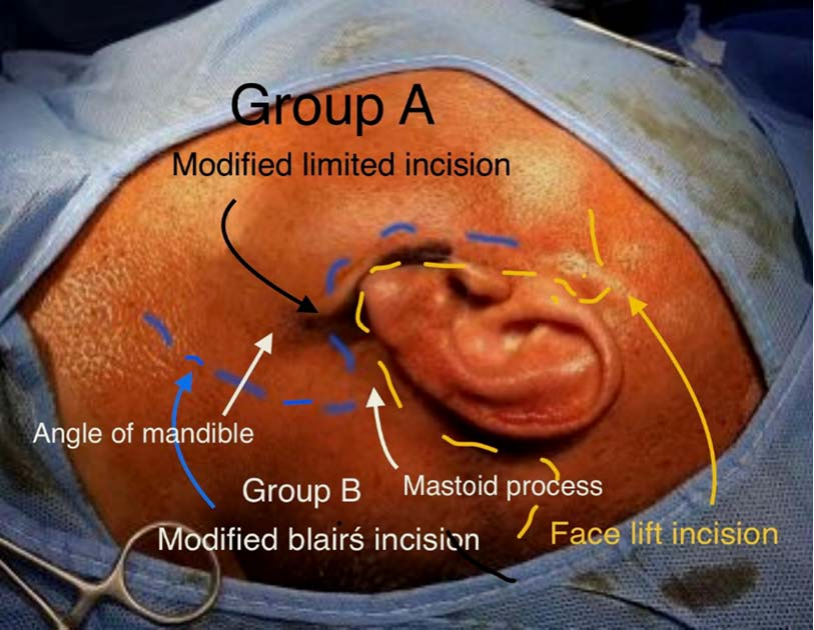
The modified limited incision used in our study.

**Figure 2:**
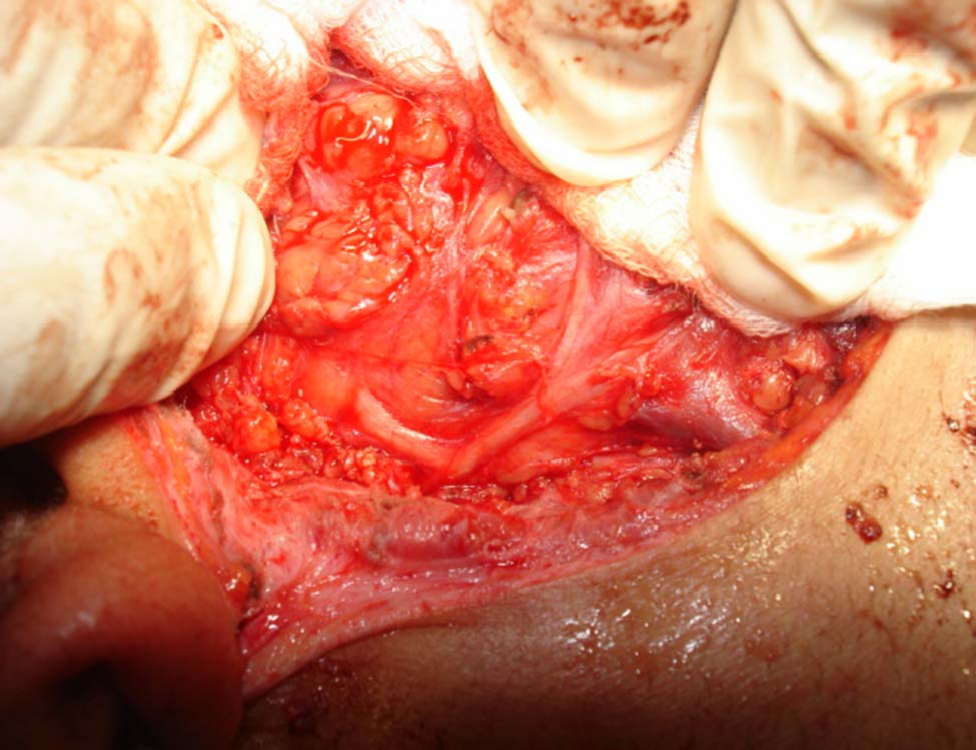
Facial nerve trunk and divisions.

**Figure 3:**
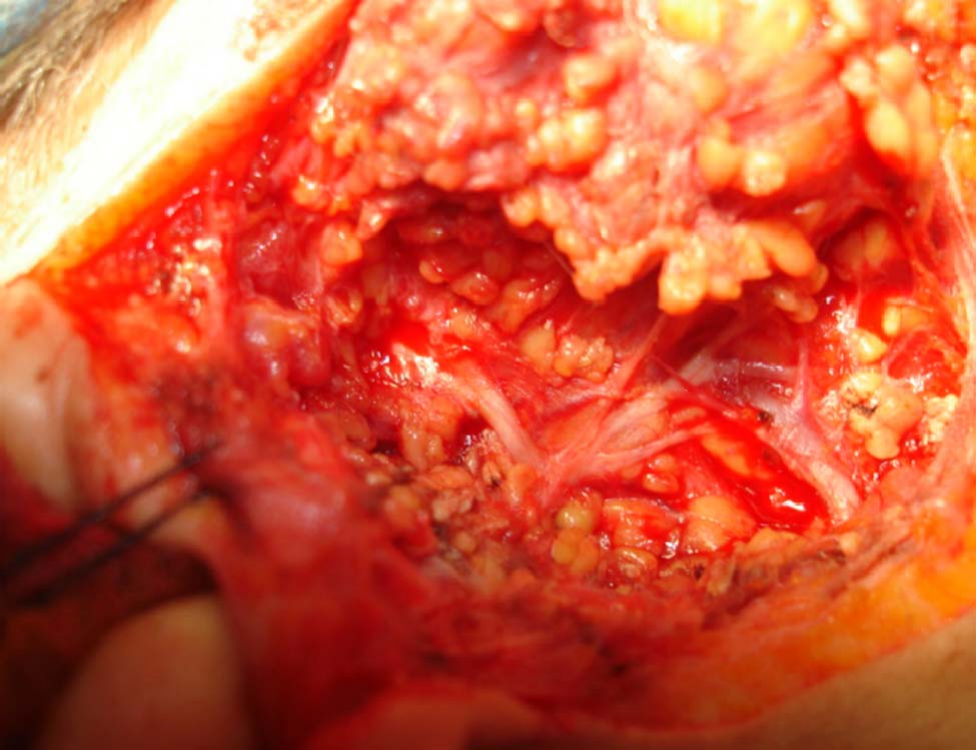
Terminal branches of the facial nerve using the modified limited incision.

**Figure 4:**
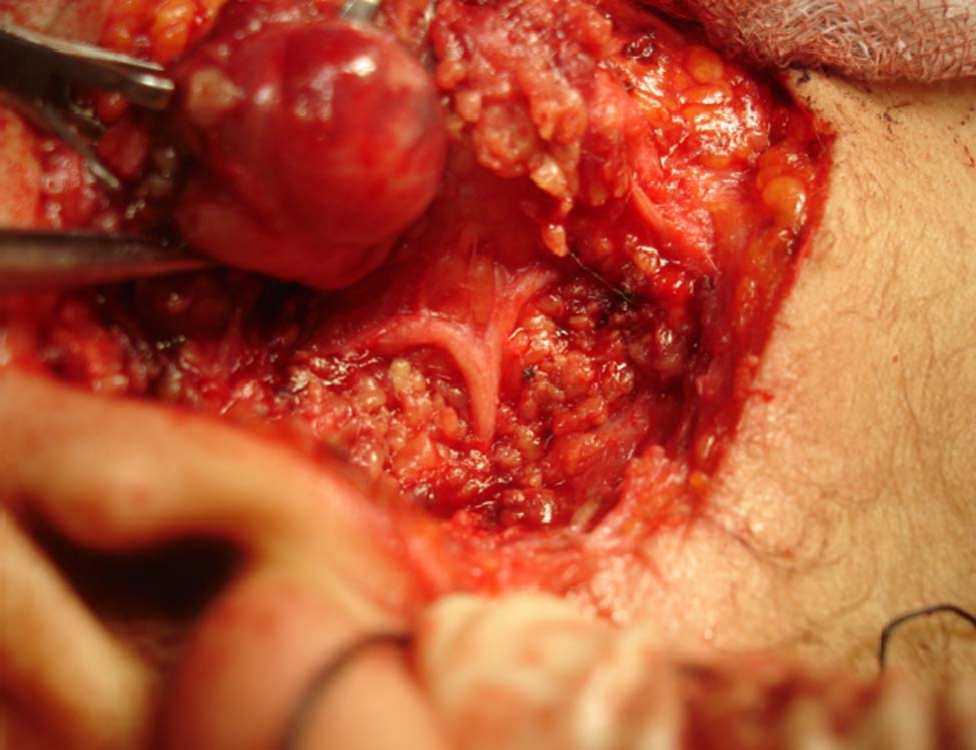
Superficial parotidectomy with the removal of the parotid tumor using the modified limited incision.

In group A, we used the modified limited incision that starts in the preauricular skin crease, extends vertically below the tragus level, continued down for 1.5 cm, then along the ear lobule, turning retroauricularly over the mastoid process, extending downwards ~1.5 cm along the inferior edge of the mandible (Fig. 1). Skin, subcutaneous tissue, and the superficial fascia were dissected and raised all around to the edge of the masseter muscle to reveal the parotid gland. The trunk of FN was localized and exposed; the divisions and terminal branches were identified (Figs 2, 3). The superficial lobe of the parotid gland was removed. The parotid duct was ligated and divided (Fig. 4). Hemostasis was secured, the wound was closed in one layer of subcuticular suturing, which was removed after 7 days. Nonabsorbable suture was used for the closure of surgical incisions depending on the surgeon’s preferences, as it is the standard method for skin closure. A Radivac drain was used. The patient was discharged from the hospital after 12 h, and the drain was removed after 48 h.

For the patients in group B, we used the same operative steps as in group A patients, the only difference was the type of incision we used which was a modified Blair’s incision, that starts as a preauricular incision below the hairline extends vertically along the anterior border of the tragus continued down for 4 cm then along the ear lobule turning retroauricularly over the mastoid process, extending downwards ~4–5 cm to follow a skin crease in the neck along with the inferior edge of the mandible (Fig. 1). Operative time was recorded for all patients. Intraoperative complications like rupture of the parotid tumor capsule, incomplete resection of the tumor, or transection of FN were not reported.

**Figure 5:**
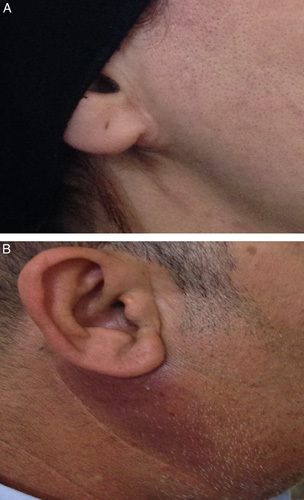
Aesthetic result of the incision after 6 months: Picture A used the modified limited incision; Picture B used the classical technique.

The follow-up of patients was as an outpatient visit after 1, 3, and 6 months, and then every year, estimating the esthetic results of the incision (Fig. 5). Patients’ satisfaction was assessed by using the visual analog scale, which was graded from 0 to 10, zero grade means least satisfied, while 10 grade means completely satisfied. Reporting early complications like hemorrhage, wound seroma or infection, FN palsy, and parotid fistula; recurrence of the tumor as a late complication. The patients’ data were entered in an Excel program.

### Statistical analyses

The data was analyzed using Statistical Package for the Social Sciences (SPSS) software version 25. Descriptive statistics were conducted for all study items. Continuous variables were expressed as means±SD, whereas categorical variables were expressed as frequencies and percentages. An independent *t*-test was used to compare the differences in the means of continuous parameters between the two study groups. Pearson *χ*^2^, and Fisher’s Exact Test were used to compare the two groups according to the categorical characteristics. A *P*-value **<**0.05 was considered as significant.

## Results

A total of 200 patients with superficial lobe parotid tumor were involved in this study. The mean age (38.0±17.2 years) was comparable between the two groups (Table 1). The majority of participating patients were male. However, the sex distribution was comparable between the two groups (Table 2). In both groups, the parotid tumor was at the right side in 56% patients, and at the left in 44% of them. In the new incision group, the tumor location within the superficial lobe was preauricular in 41%, and in the parotid tail in 59% of patients. In the new modified limited incision group, the operative time ranged from 60 to 80 min.

**Table 1:** The difference in operation time, patient age, tumor size, and satisfaction between the two study groups.

	Group	*N*	Mean	SD	*P*
Age (years)	1	100	38.02	17.24	0.980
	2	100	37.96	17.27	
Tumor size (cm)	1	100	2.47	0.83	0.972
	2	100	2.47	0.80	
Patient satisfaction	1	100	9.66	0.77	**0.000** ^*^
	2	100	8.26	1.40	
Operation time (min)	1	100	73.88	6.79	0.394
	2	100	74.75	7.60	

* Significant (*P*<0.05) according to independent *t*-test.

Group 1=new limited incision.

Group 2=control undergone modified Blair’s incision.

**Table 2:** The patient sex, tumor characteristics, and surgery complication across the two groups.

	Group		
Patient characteristics	New limited incision, *N* (%)	Control, *N* (%)	*P*
Sex			
Male	58 (58.0)	57 (57.0)	0.886
Female	42 (42.0)	43 (43.0)	
Tumor site			
Right	56 (56.0)	56 (56.0)	1.000
Left	44 (44.0)	44 (44.0)	
Tumor location			
Preauricular	41 (41.0)	41 (41.0)	1.000
Parotid	59 (59.0)	59 (59.0)	
Surgery complication			
No	95 (95.0)	94 (94.0)	0.756
Yes	5 (5.0)	6 (6.0)	

Nonsignificant (*P*>0.05) according to Pearson *χ*^2^.

Control group undergone to modified Blair’s incision.

The modified limited incision resulted in a better cosmetic outcome since it neither caused obvious scarring nor affected the FN. In the new modified limited incision group, the tumor removal without tumor capsule rupture was successful in all cases, and no further skin extension was needed. All patients were highly satisfied with the resultant appearance of the incision, and 6 months after the surgery only a nominal scar could be seen (Fig. 5). No case of skin discoloration or wound infection was seen. According to Independent *t*-tests, the satisfaction rate of the new limited incision group was significantly higher compared to those in the control B group (Table 1).

Only a small percent of the participating patients (5% in the new incision group and 6% in the control group) developed postoperative complications. The most common complication was transient FN palsy (2 out of 100 in each group) then seroma (two cases in the control group and one case in the new incision group) (Table 3). There were no statistically significant (*P*>0.05) differences between the two groups in terms of surgery time, tumor size, tumor location, indicated pathology and surgery complications (Tables 1–3). The indicated cases were comparable between the two groups in terms of histopathological findings. In the new incision group, the histopathological findings were pleomorphic adenoma (69%), Warthin’s tumor (28%), monomorphic adenoma (2%), and hemangioma (*n*=1%) (Table 4).

**Table 3:** Postoperative complications of the patients in the two groups.

	Group		
Surgery complications	New limited incision, *N* (%)	Control, *N* (%)	*P*
No complication	95 (95.0)	94 (94.0)	
Transient facial nerve palsy	2 (2.0)	2 (2.0)	
Hypoesthesia of greater auricular nerve	1 (1.0)	1 (1.0)	
Seroma	1 (1.0)	2 (2.0)	0.987
Parotid fistula	1 (1.0)	1 (1.0)	
Total	100 (100.0)	100 (100.0)	

Nonsignificant (*P*>0.05) according to Fisher’s exact test.

Control group undergone to modified Blair’s incision.

**Table 4:** The pathological types of tumors across the patients in the two groups.

	Group		
Tumor pathological type	New limited incision, *N* (%)	Control, *N* (%)	*P*
Pleomorphic adenoma	69 (69.0)	70 (70.0)	1.000
Warthin’s tumor	28 (28.0)	28 (28.0)	
Monomorphic adenoma	2 (2.0)	2 (2.0)	
Hemangioma	1 (1.0)	0 (0.0)	

Nonsignificant (*P*>0.05) according to Fisher’s exact test.

## Discussion

Searching for a minimally visible scar is an aim for any surgeon. The modified Blair incision is used by many surgeons to perform parotidectomy^16,17^. Rhytidectomy (face-lift incision) by Appiani and Delfino^17^ has become very popular as it provides good exposure with a more cosmetic appearance than Blair’s incision^18^. Shemen *et al*.^19^ used a modified rhytidectomy incision consisting of a preauricular and postauricular portion, which avoids the hairline scar. Access to the parotid gland is sometimes difficult with the face-lift incision. The Blair incision and its modifications may lead to skin discoloration around the ear lobule.

In our modified incision there was excellent healing, no extra curvature, the tumor access was adequate, maintained blood supply of the flap. Cosmetically acceptable due to no visible scar.

Using other incisions required a longer operating time as reported by other studies. Pages *et al*.^5^ reported an operative time ranging from 70 to 180 min. In our study, the operative time was shorter, 60–80 min, which may be explained by the inclusion of only small size tumor of benign pathology and only superficial parotidectomy, also our own experience in parotid surgery as we do 2–3 surgeries a week, while others involved different types of parotidectomy (superficial and total parotidectomies) in their study^5,20^. In our study, in some patient’s in group B, the operative time was longer because of the longer wound, which required more time for hemostasis, and closure.

Regarding postoperative complications, our results concerning transient FN palsy was close to that reported by Pages *et al*.^5^, and Alomar OSK^21^ via the periauricular incision, while Shaaban and Abdelmohsen^20^, and Ahn *et al*.^22^ recorded a lower incidence of postparotidectomy transient FN palsy via the periauricular incision. However, permanent FN palsy was not reported and this is comparable to most of the authors who used the periauricular incision for parotidectomy^21–25^.

Petroianu^4^ reported three cases of permanent FN palsy (3/39), total parotidectomy was done for all these patients via the periauricular incision for malignant tumors. We thought the complications were related to the procedure itself and not related to the incision type.

The incidence of other postoperative complications (sialocele, hematoma, and Frey’s syndrome) was comparable to others^20,21,23^.

In the present study, all parotidectomies were done successfully by the limited incision without further skin extension. In contrast, other authors reported the need for extension of their limited incision in some cases^20,22^. The reason may be the position of our limited incision is more feasible, and it can be extended as required.

We assessed patients satisfaction by using a visual analog scale, which proved to be reliable, valid, and feasible. This method for scar assessment is highly sensitive and capable of reliably measuring differences in scar quality, making them valuable techniques. Combining logistic regression with an area under the curve of 0.72 in a receiver operating characteristic curve analysis, the interrater reliability of the overall scale showed an intraclass correlation coefficient of 0.95 (95% CI, 0.96–0.99) using a two-sample random-effects model. The visual analog scale score was shown to be a highly statistically significant predictor of a good scar^26,27^. Scar scales allow the assessment of additional health domains that cannot be observed by clinicians, such as scar sensations, symptoms, and quality of life. It is a fast evaluation of multiple scar characteristics, free, and easily applied.

Patients’ quality of life was adversely affected after classical surgery of the parotid gland because of the evident scar. Thus, many patients refused doing the operation till their tumor becomes very big in size or transformed into a malignant one, because they are afraid from the cosmetic appearance of the wound scar. That is why we were encouraged to do this study.

The main strength of the present study was the availability of the comparative group, and prospective in nature. However, it had some limitations such as the inclusion of patients with benign tumors only, confined to the superficial lobe, with a size less than 4 cm. In the future, we recommend using this limited incision for different types of parotidectomy, and in different locations of parotid tumors.

## Conclusion

In conclusion, superficial parotidectomy can be performed safely via our limited incision with a better esthetic outlook of the surgical scar compared with the modified Blair’s incision. We recommend more studies with a larger number of patients to estimate our incision for different types of parotidectomy and for different parotid tumors locations.

## Ethical approval

The approval of the ethics committee was obtained from Health Ethics Committee in College of Medicine, University of Baghdad, with a registration number: 44. Ethical considerations were obtained according to Helsinki Declaration.

## Written informed consent

Written informed consent was obtained from the patient for publication and any accompanying images. A copy of the written consent is available for review by the Editor-in-Chief of this journal on request.

Parental Consent for Minors: Please include as follows: Written informed consent was obtained from the patient's parents/legal guardian for publication and any accompanying images. A copy of the written consent is available for review by the Editor-in-Chief of this journal on request.

## Sources of funding

I declared all sources of funding. And declare the role of study sponsors, if any, in the collection, analysis, and interpretation of data; in the writing of the manuscript; and in the decision to submit the manuscript for publication.

## Author contribution

Single author, did the study concept or design, data collection, data analysis or interpretation, and writing the paper.

## Conflicts of interest disclosure

There are no conflicts of interest.

## Research registration unique identifying number (UIN)

1. Name of the registry: http://www.researchregistry.com
2. Unique identifying number or registration ID: Researchregistry 8594.
3. Hyperlink to registration: https://www.researchregistry.com/browse-the-registry#home (Researchregistry 8594. Available at: https://www.researchregistry.com https://www.researchregistry.com/browse-the-registry#home).

## Guarantor

No guarantor.

## Data availability statement

Data sharing is not applicable to this article.

## Provenance and peer review

Not commissioned, externally peer reviewed.
